# Label-free macrophage phenotype classification using machine learning methods

**DOI:** 10.1038/s41598-023-32158-7

**Published:** 2023-03-30

**Authors:** Tetiana Hourani, Alexis Perez-Gonzalez, Khashayar Khoshmanesh, Rodney Luwor, Adrian A. Achuthan, Sara Baratchi, Neil M. O’Brien-Simpson, Akram Al-Hourani

**Affiliations:** 1 Department of Medicine, Royal Melbourne Hospital, The University of Melbourne, Parkville, VIC 3050 Australia; 2 Melbourne Cytometry Platform, Department of Microbiology and Immunology, The University of Melbourne, at The Peter Doherty Institute of Infection and Immunity, Parkville, VIC 3010 Australia; 3 School of Engineering, RMIT University, Melbourne, Victoria 3000 Australia; 4 Department of Surgery, Royal Melbourne Hospital, The University of Melbourne, Parkville, Victoria 3050 Australia; 5 Fiona Elsey Cancer Research Institute, Ballarat, Victoria 3350 Australia; 6 Federation University Australia, Ballarat, Victoria 3350 Australia; 7 School of Health & Biomedical Sciences, RMIT University, Bundoora, Victoria 3083 Australia; 8 ACTV Research Group, Division of Basic and Clinical Oral Sciences, Centre for Oral Health Research, Melbourne Dental School, Royal Dental Hospital, The University of Melbourne, 720 Swanston Street, Carlton, VIC 3010 Australia

**Keywords:** Biological techniques, Cell biology, Immunology

## Abstract

Macrophages are heterogeneous innate immune cells that are functionally shaped by their surrounding microenvironment. Diverse macrophage populations have multifaceted differences related to their morphology, metabolism, expressed markers, and functions, where the identification of the different phenotypes is of an utmost importance in modelling immune response. While expressed markers are the most used signature to classify phenotypes, multiple reports indicate that macrophage morphology and autofluorescence are also valuable clues that can be used in the identification process. In this work, we investigated macrophage autofluorescence as a distinct feature for classifying six different macrophage phenotypes, namely: M0, M1, M2a, M2b, M2c, and M2d. The identification was based on extracted signals from multi-channel/multi-wavelength flow cytometer. To achieve the identification, we constructed a dataset containing 152,438 cell events each having a response vector of 45 optical signals fingerprint. Based on this dataset, we applied different supervised machine learning methods to detect phenotype specific fingerprint from the response vector, where the fully connected neural network architecture provided the highest classification accuracy of 75.8% for the six phenotypes compared simultaneously. Furthermore, by restricting the number of phenotypes in the experiment, the proposed framework produces higher classification accuracies, averaging 92.0%, 91.9%, 84.2%, and 80.4% for a pool of two, three, four, five phenotypes, respectively. These results indicate the potential of the intrinsic autofluorescence for classifying macrophage phenotypes, with the proposed method being quick, simple, and cost-effective way to accelerate the discovery of macrophage phenotypical diversity.

## Introduction

Macrophages are innate immune cells that are highly responsive to their surrounding microenvironment^1^. According to the microenvironmental signals, macrophages adapt phenotypically to perform multiple beneficial functions required for body survival, like tissue surveillance, remodeling, and defense^2,3^. On the other hand, the tissue microenvironment can influence macrophages to promote various pathologies like cancer^4^, infections^5^, rheumatoid arthritis^6^, atherosclerosis^7^, and severe COVID-19^8^. Current understanding of what factors are responsible for the generation of disease-protective and disease-promoting macrophage phenotypes is limited^5,9^. Therefore, the investigation of macrophage responsiveness to various biomolecules and therapeutic compounds is a key mechanism to understand macrophage phenotypical diversity and how it can be therapeutically manipulated to treat pathologies.

Currently, the complexity of macrophage phenotypical diversity is extremely simplified by dividing macrophage phenotypes according to their functional characteristics^10^, expressed markers^11^, and polarization agents^12^. M1-M2 dichotomy is the most common way to define macrophage phenotypes that can be reproduced in vitro and it includes M0 (naïve), M1, M2a, M2b, M2c, and M2d phenotypes^11,13–18^. However, this M1-M2 macrophage classification lacks in vivo applicability as it is broadly recognized by multiple studies that regularly reporting novel macrophage phenotypes^4,19,20^. Macrophage phenotypic diversity is usually investigated by applying various biological detection techniques such as flow cytometry, immunohistochemistry, qPCR, mRNA sequencing, and Western blotting^9,21^. While being well-established and informative, these techniques are also time-consuming and costly as each of these methods require a scrupulous and lengthy experimental planning, sample preparation, and processing. In addition, there are several limitations due to the incomplete knowledge of cell specific markers and biological assays that are also limited in their detection capabilities, creating challenges in detecting low expressed markers^31^. Thus, there is a real need for the continuous development of supplementary methods that could potentially be simpler, cost effective, quicker, and accurate in validating macrophage responses to their microenvironments.

Potential methods to investigate macrophage populations include the exploration of morphology and intrinsic cell characteristics, that are known to be affected by different treatments^22–25^. For example, Rostam et al.^22^ developed an image-based machine learning method with 90% accuracy in discrimination of M1 and M2 macrophages from naïve monocytes and macrophages by their morphological characteristics, Li et al.^23^ was able to discriminate M1, M2a, M2b, M2c macrophages based on their mitochondria organization, and Geng et al.^26^ reported unique fluorescent fingerprints in M1(IFN-γ), M1(LPS), M1(IFN-γ + LPS), M2a (IL-4), and macrophages polarized in the presence of tumor conditioned media upon the interaction of macrophage cell membrane with polymer–protein sensor C3-Gu-Py + GFP^26^. In order to achieve classification, the above-mentioned studies used fluorescent dyes to observe cell morphological features. On the contrary, label-free methods to investigate macrophages has also been developed. For example, applying machine learning, Pavillon et al.^27^ combined macrophage morphology and Raman spectroscopy readings to detect six different activation status in label-free LPS-treated macrophages exposed to dexamethasone with accuracy of 84–87%. Label-free data was compared to the traditional detection methods such as tissue necrosis factor (TNF) assay and iNOS immunofluorescent staining. Indeed, Raman spectrometry is a very powerful tool that shown to be useful in cell classification, however its efficiency might be affected by cell autofluorescence signal interfering with Raman scattering which is an inherently weak phenomenon^28^ and experimental results can also be affected by sample preparation technique^29^. Raman spectroscopy typically requires samples to be in a thin, homogeneous layer to maximize the Raman signal which might be challenging for label free investigation of delicate samples^30^. Macrophage autofluorescence also demonstrated to be a unique signature allowing macrophage label-free detection. For example, Rico-Jimenes et al.^24^ used macrophage autofluorescence to detect macrophage accumulation in atherosclerotic plaque using lifetime imaging achieving 91.5% accuracy when compared to CD68 macrophage staining and Heaster et al.^25^ showed that intravital metabolic autofluorescence imaging was able to identify macrophage heterogeneity and tissue-specific metabolic behavior in vivo confirming results with immunofluorescent staining post mortem. These studies provide evidence that autofluorescence can be utilized to extract cell specific information being technically simpler, non-invasive, and importantly, non-destructive to samples that enables further sample usage applying traditional techniques if required.

In addition to microscopy and imaging, single cell autofluorescence can be investigated by flow cytometry. Flow cytometry is a well-established and valuable tool to analyze individual cells, thanks to its ability to detect cell morphology, viability, cell cycle, and expressed surface and intracellular markers^31^. Modern flow cytometers are equipped with multiple excitation lasers and can record emission signal across multiple narrow band optical filters. Out of the optical signal measured at each detector, flow cytometer signal is usually characterized using three parameters corresponding to (i) the signal pulse area, (ii) height, and (iii) width. Traditionally, the analysis of polychromatic flow cytometry experiments involves the visualization of each fluorescent marker across histograms or bivariate plots based on one property of the signal pulse (Area or Height) out of a single detector (corresponding to the fluorochrome emission maximum). The identification of unique cellular subsets is commonly achieved via manual hierarchical two-dimensional gating and comparisons against unstained samples, single stained and experimental controls. As an alternative to this traditional approach, all recorded signals associated with each cell can be combined in a multidimensional data array^31,32^ which can further undergo a computational analysis for signal interpretation^31^. Such multidimensional analysis can potentially carry the unique autofluorescence fingerprint for label-free samples and be further explored in cells detection.

Machine learning is a promising method to analyze complex signals data^33^ and has been shown to be a powerful tool in many biological applications, including label-free histology^34^, label-free imaging flow cytometry^35,36^, and label-free fluorescence-activated cell sorting^37^. Machine learning has long been applied to accelerate the biomedical research as it can automatically analyze large datasets to identify patterns and make automated decisions^38,39^. Two main approaches in machine learning are: (i) unsupervised machine learning^40^ that identifies de novo patterns without the need for manually labelling the training dataset, this is usually applied when it is impossible (or impractical) to segregate and label the dataset, (ii) supervised machine learning^41,42^ where machines are trained using labelled data set with the desired outcome; then the machine is supposed to reasonably recognize similar patterns that have not been shown to it previously. In particular, as a subset of machine learning, artificial neural networks have increasingly been investigated in many scientific fields due to their superior performance against traditional manual and threshold-based methods^41,42^. This has been in-part due to the increasing computational power and the advent of powerful graphical processing units (GPUs) in addition to the new machine learning algorithms that made this leap feasible.

Accordingly, this paper investigates phenotype-specific autofluorescence as a clue for classifying different macrophage phenotypes. For this purpose, we have generated six macrophage phenotypes and applied a neural network to analyze macrophage autofluorescence collected during a flow cytometry experiment. The trained network was successfully able to detect the class, i.e., phenotype, of the testing set with reasonable accuracy. In this way we aim to present a framework to classify different macrophage phenotypes based on their unique autofluorescence signature. This framework provides a simple, time- and cost-effective method that can augment existing methods in studies exploring cell populations and investigating cellular response to various stimuli. Our work provides a method to extract a multi-dimensional autofluorescence signature from a conventional flow cytometer with further analysis applying machine learning that allows a successful classification of six macrophage phenotypes.

## Results

**Figure 1:**
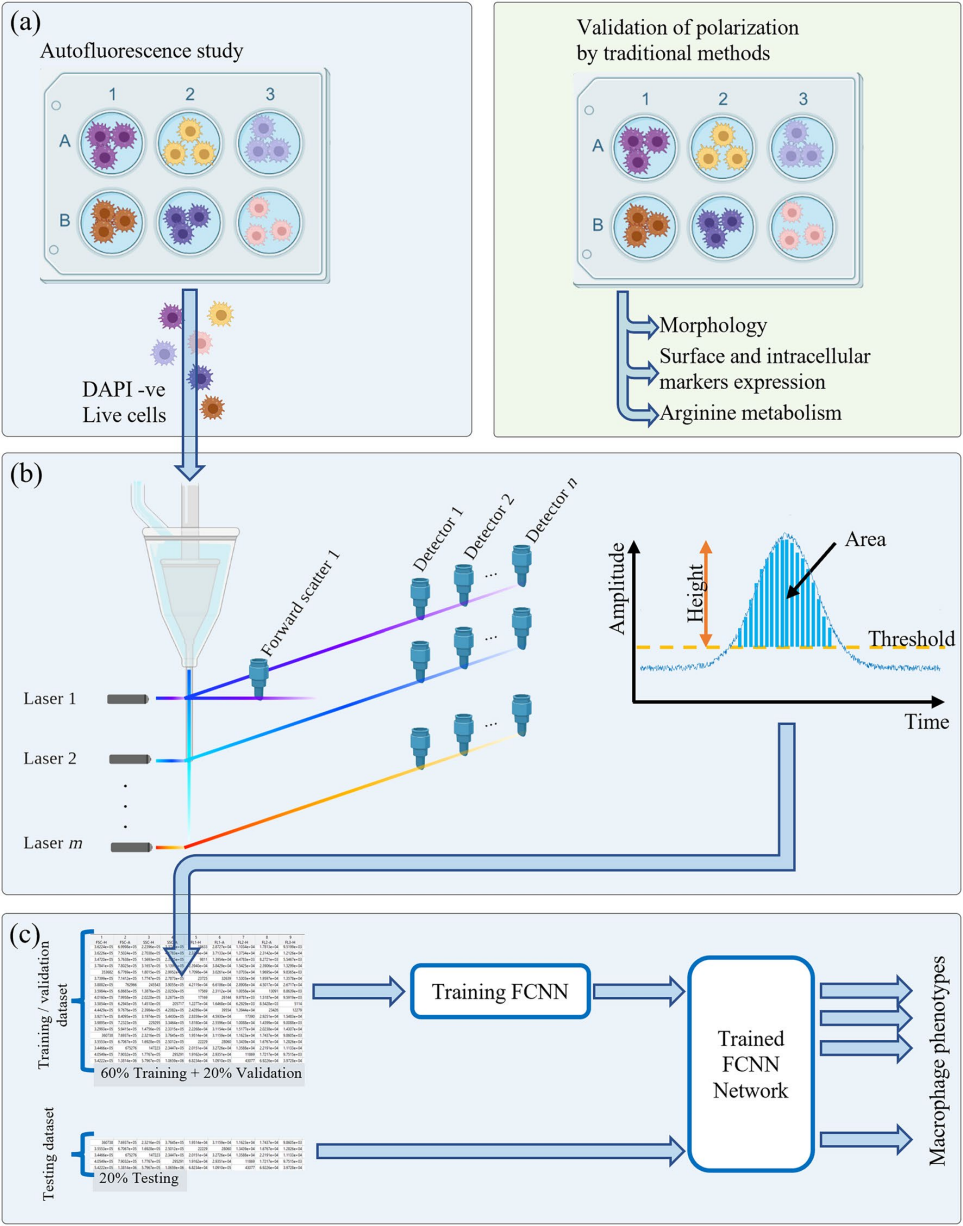
Workflow of the macrophage intrinsic signal analysis. (**a**) Right block: six macrophage phenotypes were induced by applying different stimuli and macrophage polarization confirmed by measuring biological characteristics validated in accordance with previous studies. (**a**) Left block: a separate macrophage polarization experiment was used to investigate the autofluorescence signature for each phenotype. DAPI staining was used to exclude dead cells and autofluorescence of live cells was measured. (**b**) Label free polarized (DAPI-/live) macrophage samples were acquired in a Beckman Coulter CytoFLEX LX flow cytometer recording 45 parameters per cell = 20 fluorescence detector × 2 (height and area) + forward scatter 1 × 3 (height, area, width) + side scatter 1 × 2 (height and area). (**c**) The generated data was divided into three parts: training (60%), validation (20%), and testing (20%), where a supervised fully connected neural network (FCNN) classified the different phenotypes based on cell specific signatures.

We developed a series of experiments where six macrophage phenotypes were cultured and polarized according to well-known protocols in the literature^3,11,43–45^. The polarization was validated by four traditional methods: (i) immunostaining, (ii) polychromatic flow cytometry, (iii) Griess assay, and (iv) arginase assay, as indicated in Fig. 1a. As such, the status of macrophage polarization was known prior to the investigation of autofluorescence. A parallel polarization experiment was prepared to contain cells that are labelled with only a viability dye DAPI in order to segregate living cells; where dead cells are excluded from the analysis.

Accordingly, we recorded the autofluorescence signal of the viable six macrophage phenotypes by a conventional flow cytometer equipped with six lasers (each having different emission wavelength). The emitted autofluorescence from each optically interrogated cell was collected from all available narrow band optical filters (Fig. 1b). In total, 45 parameters per cell were collected including: (i) height, area, and width values from the forward scatter detector; (ii) height and area from the side scatter detector; (iii) 40 signals from 20 fluorescence detectors (height and area). In total, this experiment generated 152,438 cell events for the six phenotypes, among which 60% of data were used for *training* a fully connected neural network (FCNN), 20% were used during the *validation* process, and the remaining 20% were used for network *testing* (Fig. 1c), this last portion did not contribute in the training process and thus used to judge the resulting performance. The details of the utilized methods for both experiment and data analysis are provided in section "Detailed methods and data analysis".

In addition, we would like to highlight that the term “labelling” is used differently in the field of data science and the field of biology. In this paper the term “labelling” has a biological meaning and refers to the process of attaching a chemical or molecular tag (antibody, dye) to a specific type of cell, which is used to identify these cells in various biological and medical applications.

### Macrophage polarization validation

To construct our dataset, six macrophage phenotypes namely M0 (unpolarized—naïve), M1, M2a, M2b, M2c, and M2d were induced by applying various polarization agents. Macrophage polarization was validated using four established laboratory techniques, namely: (i) immunostaining, (ii) flow cytometry, (iii) Griess assay, and (iv) arginase assay. The investigated phenotypical characteristics were selected based on these previous studies^3,11,43–45^.

**Figure 2:**
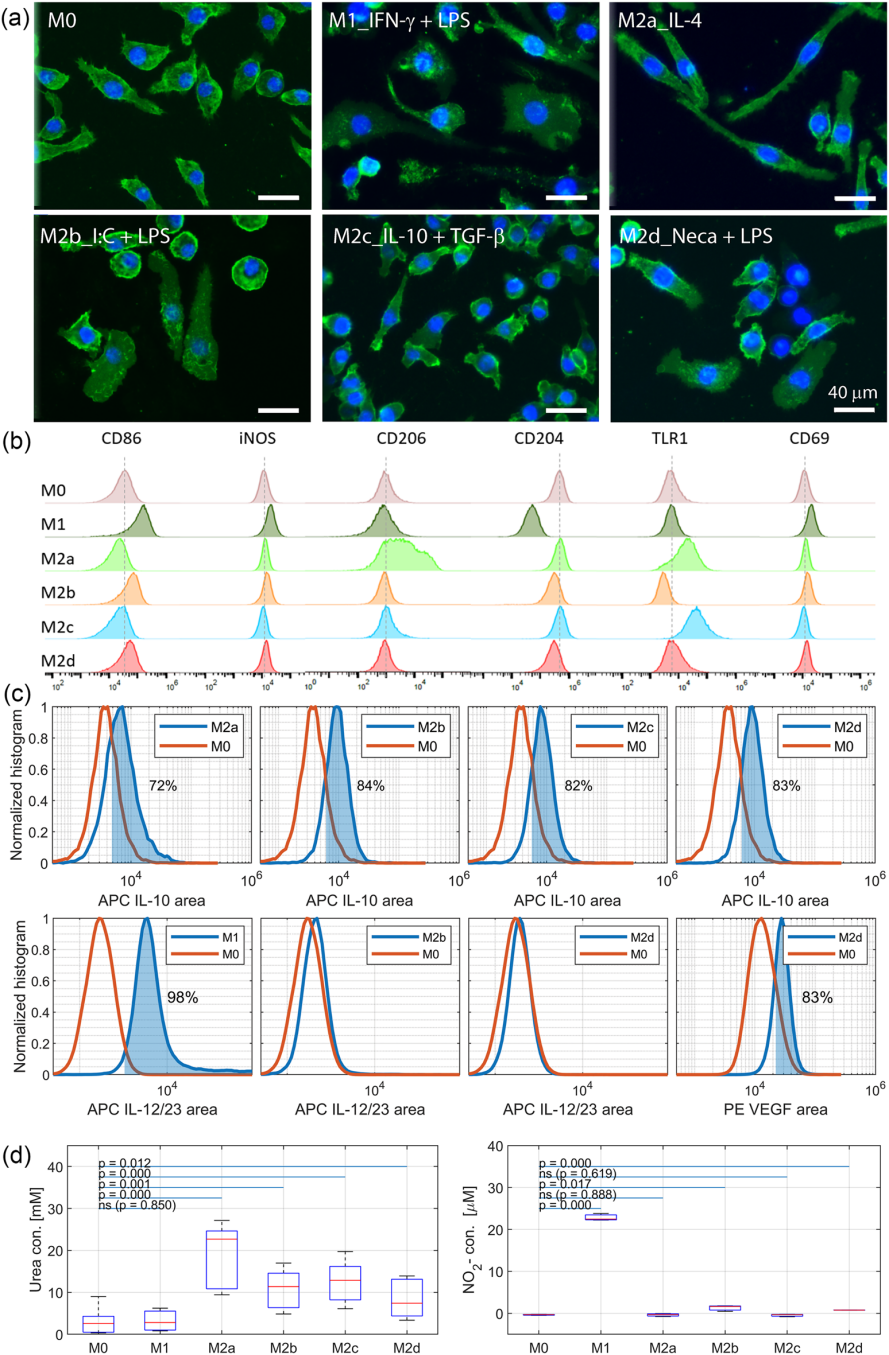
Unpolarized and polarized RAW264.7 macrophages displayed different morphology, expression markers, and metabolic activity. (**a**) Morphology of M0, M1, M2a, M2b, M2c and M2d macrophages with cell membrane labelled with PKH67 (green) and cell nuclei with DAPI (blue), scale bar 40 μm. (**b**) Surface and intracellular expression of CD86, iNOS, CD206, CD204, TLR1, and CD69. (**c**) IL-10, IL-12, and VEGF expression. (**d**) Arginine metabolic activity of the polarized macrophages, data are representative of three separate experiments showing the mean ± standard error of mean (SEM).

Under the microscope, polarized macrophages showed different morphology compared to unpolarized. M0 were morphologically heterogeneous cell populations with round and slightly elongated cells, M1 polarized cells appeared larger, and flattened with irregular shape and extended pseudopods. These cells occasionally appeared as multinucleated due to the interferon gamma (IFN-γ) induced cells fusion as previously described^46^. M2a macrophages had elongated and narrowed shape, M2b macrophages were flattened, elongated, and granular. M2c macrophages were morphologically similar to M0, however appeared more circular and slightly smaller. Finally, M2d macrophages were flattened and elongated (Fig. 2a).

As per Fig. 2b, c, polarized macrophages also differed in marker expression. Following differentiation with IFN‐γ and LPS, M0 macrophages displayed the typical M1 macrophage phenotype (CD86^high^, iNOS^+^, CD69^+^, and IL-12/IL-23^+^). Similarly, the incubation of M0 with interleukin 4 (IL-4) induced M2a phenotype (CD206^high^, CD204^+^, TLR1^+^, and IL-10 +); incubation with immune complexes (I:C) and LPS induced M2b phenotype (CD86^+^, iNOS^+^, CD69^+^, IL-10^+^, and IL-12/IL-23^-^); IL-10 and transforming growth factor beta (TGF‐β) stimulation induced M2c phenotype (CD204^+^, TLR1^+^, and IL-10^+^); and 5'-N-Ethylcarboxamidoadenine (NECA) with LPS induced M2d phenotype (CD86^+^, iNOS^+^, IL-10^+^, VEGF^+^, and IL-12/IL-23^-^).

M1 and M2 macrophages are known to metabolize arginine by two different pathways^40^, which were investigated by arginase assay and Griess assay. As per Fig. 2d, six macrophage subtypes showed different arginine metabolic activities. Upon stimulation, M1, M2b and M2d phenotypes upregulated NO production with p-values (p = 0.000, p = 0.017, and p = 0.000, respectively), while urea production was seen in M2a, M2b, M2c, and M2d with p-values (p = 0.000, p = 0.001, p = 0.000, and p = 0.012, respectively). Overall, the collected results validated the polarization of M0 macrophages into the five macrophage phenotypes and the same polarization conditions were used to conduct the flow cytometry experiment with the label free cells to investigate phenotype specific autofluorescence.

### Label free macrophages signature

The hypothesis of this study was that the signal recorded by the conventional flow cytometer (CytoFLEX LX) across all detectors collectively could generate a unique fingerprint for each macrophage phenotype. To verify our hypothesis, we included only viable cells in our analysis, as dead cells possess a broad spectrum of autofluorescence that could interfere with that from live cell autofluorescence^47^. To exclude dead cells, DAPI staining was used at low dye concentration to prevent the staining of living cells.

**Figure 3:**
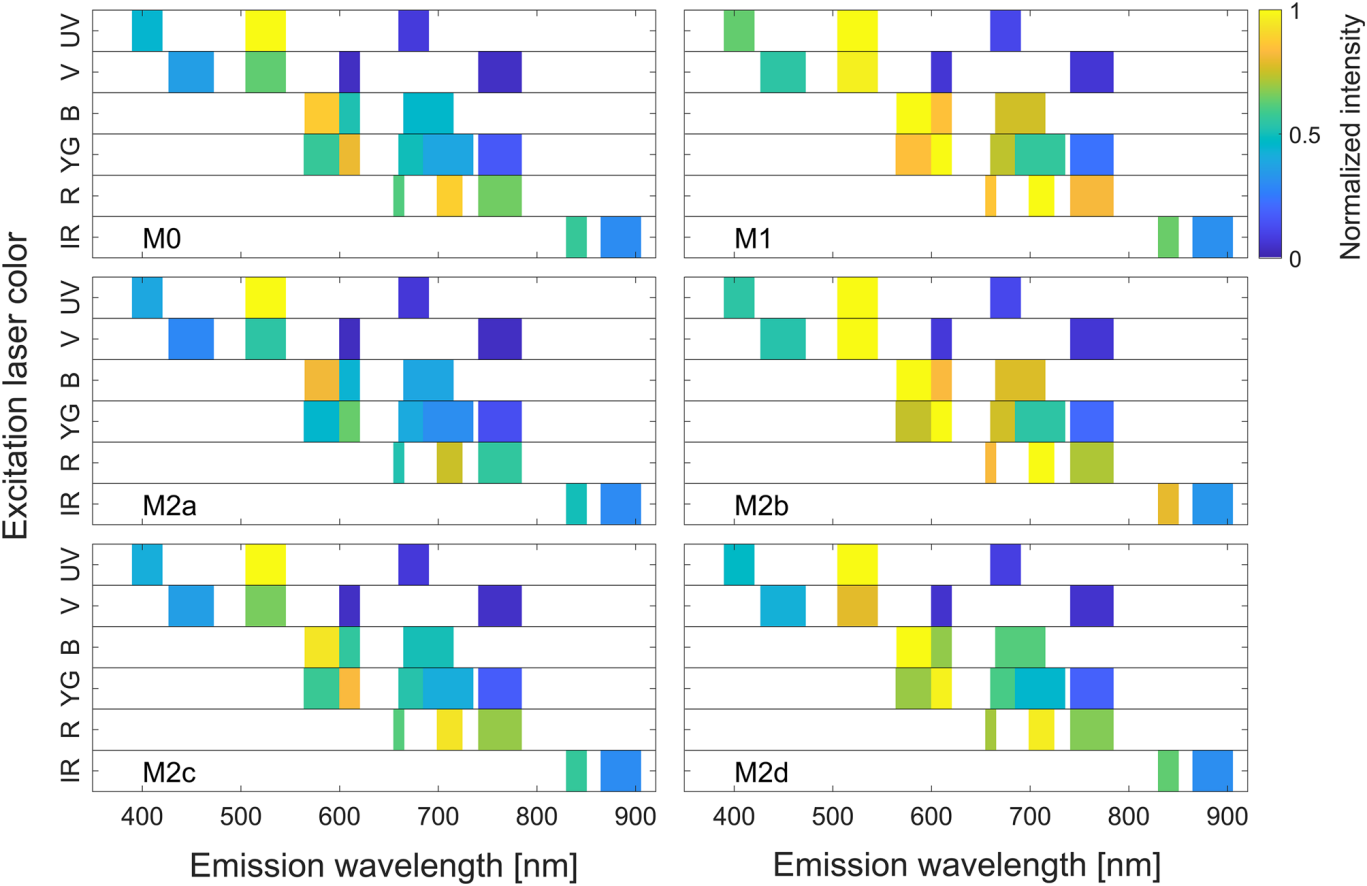
An intensity plot for the average height of the forward scatter signal for each macrophage phenotype. The signal is collected across all available optical detectors upon exposure to infra-red (IR), red (R), yellow/green (YG), blue (B), violet (V), and ultraviolet (UV) lasers at emission wavelengths 808 nm, 638 nm, 561 nm, 488 nm, 405 nm, respectively. The intensity is normalized to the same scale across all phenotypes. Each colored block represents the collected signal per each detector generated after excitation with the corresponding laser.

**Figure 4:**
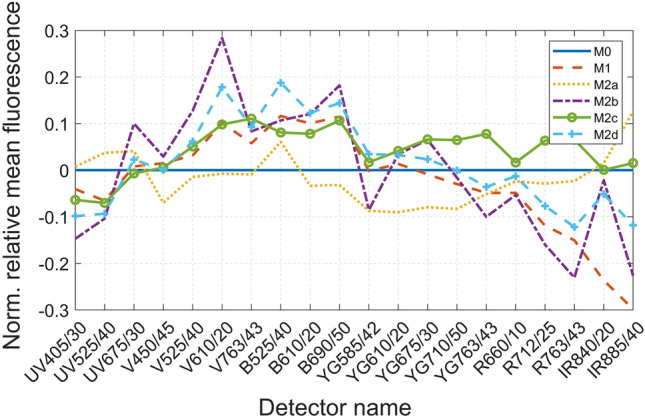
Comparison of the mean fluorescence intensity plot for the pulse height across the six phenotypes. This represents the same data in Fig. 3, however the intensities are normalized to M0 phenotype. The naming convention of the detectors is $C\lambda \lambda \lambda /\beta \beta$, where *C* represents the laser color, $\lambda \lambda \lambda$ represents the detector’s wavelength, and $\beta \beta$ represents the detector’s bandwidth (measured in nm).

In total, 152,438 live cell events were recorded in the flow cytometry experiment. Firstly, after signal normalization, an intensity plot was generated that showed the fingerprint (i.e., signal intensity across all detector channels) for each phenotype. Since the feature vector is composed of 45 signals, we only show an example in Fig. 3 based on the average pulse height signatures (representing the mean autofluorescence intensity) of the six macrophage phenotypes collected across all optical detectors. We note from this figure, as an example, the apparent similarity between M0 and M1 in the infrared laser channels, however, by looking at the red laser detectors we can observe different intrinsic signals. A visual inspection of the signals indicates that each phenotype has a unique autofluorescence fingerprint, however with the data of such high dimensionality (i.e., 45 signals) it is impractical to manually quantify these mutual differences among the macrophage populations, instead computerized algorithms have a greater accuracy and are far more efficient in dealing with such high dimensional signatures. To better visualize the relative autofluorescence differences between the six phenotypes, we depict in Fig. 4 the normalized mean intensity (signal height) obtained across the 20 detectors, where the normalization is performed with respect to the mean intensity of M0. In particular, the normalization is performed into two stages: (i) in the first stage we normalize each phenotype across all detectors to suppress broadband intensity variations as follows,

$${\overline{y} }_{ij}=\frac{{y}_{ij}}{\sum_{j=0}^{20}{y}_{ij}},$$

where ${y}_{ij}$ represents the mean intensity of all the samples for phenotype $i$ and detector $j$, and ${\overline{y} }_{ij}$ is the first stage normalized vector. (ii) In the second stage the normalization is performed with respect to M0 phenotype as follows,

$${\widetilde{y}}_{ij}=\frac{{\overline{y} }_{ij}-{\overline{y} }_{1j}}{{\overline{y} }_{1j}},$$

where ${\overline{y} }_{1j}$ represents the first stage normalized mean responses of M0 phenotype. Accordingly, the M0 mean intensity appears as a horizontal line at unity in Fig. 4. We note based on this figure the significant differences across multiple detectors with a rich signature profile distinguishing the different phenotypes. This complex signature of the heights along with the remaining 25 parameters are used as clues for the neural network to perform the classification as it will be explained in the next subsection.

### Macrophage classification accuracy applying machine learning

**Figure 5:**
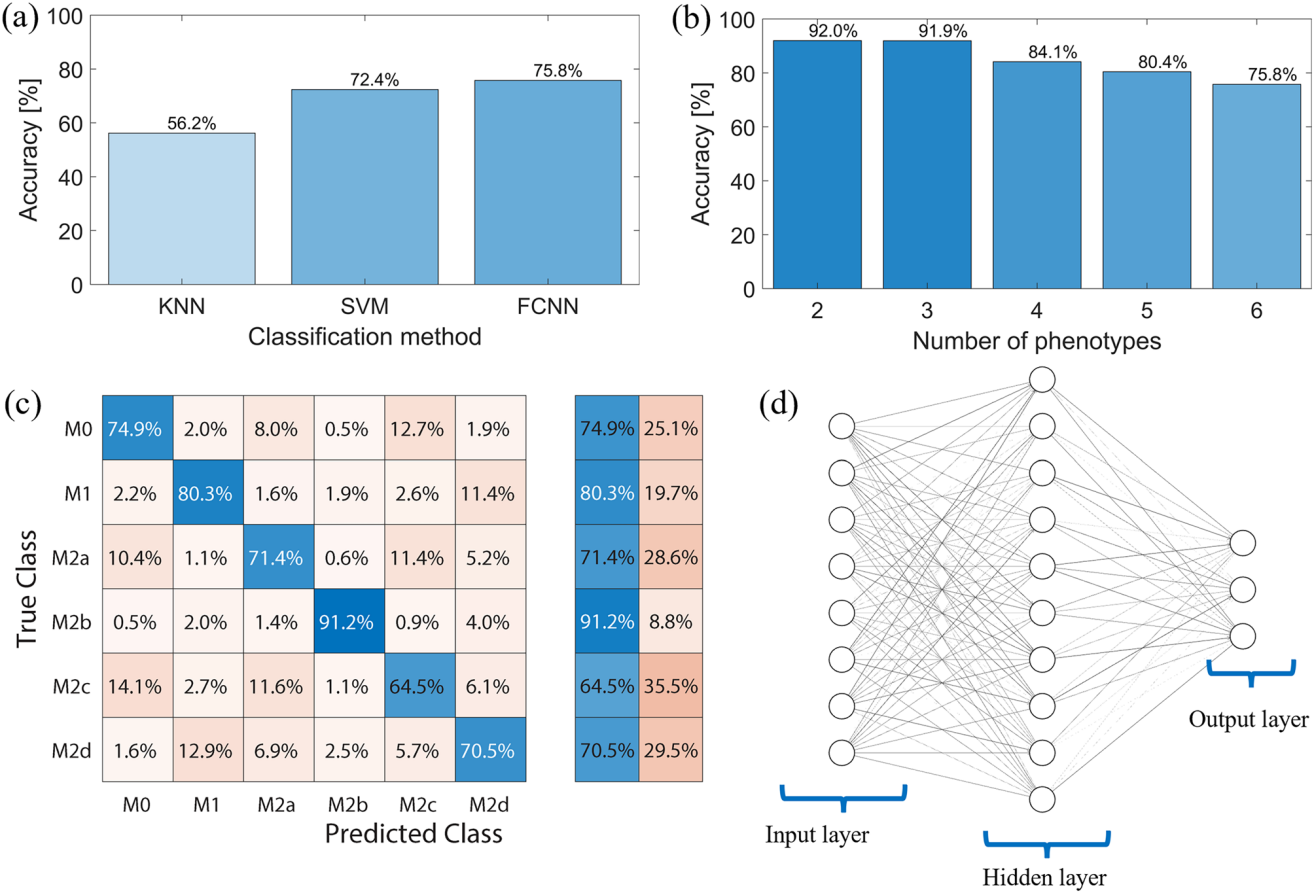
Label-free macrophage classification accuracy. (**a**) Classification accuracy using KNN, SVM and FCNN classifiers tested for their ability to classify the six macrophage phenotypes, where the FCNN is noted to provide the highest classification accuracy. (**b**) The classification accuracy of any 2, 3, 4, 5 phenotypes combinations, comparing to the 6 phenotypes. (**c**) The confusion matrix representing the accuracy of macrophage phenotyping using intrinsic autofluorescence. In blue are the correctly identified cells, while phenotypes not correctly identified are in pale red. Difference in color intensity is indicative for numerical values with darker shades representing higher values. The table on the right demonstrates overall summary for correctly and not correctly identified cells (i.e., true positives and false positive). (**d**) A representation of the utilized Fully Connected Neural Network (FCNN) architecture with a single wide hidden layer. The number of neurons in the figure are for illustration only, the actual utilized number is 45 for the input, 100 for the hidden layer, and 6 for the output as it will be further explained in the “Detailed methods and data analysis” section.

After collecting the cellular fingerprints, the collected data was randomly divided as follows: 60% of data were used for network training, 20% of data were used for validation, and 20% of data were used for testing. Then, these signatures were analyzed by multiple classifiers, however, three different machine learning methods showed the best results, namely: (i) *k*-nearest neighbor (KNN) with 50 neighbors^48^, (ii) support vector machine (SVM)^49^, and (iii) fully connected neural network (FCNN)^50^. Among these methods, FCNN demonstrated the highest classification accuracy of 75.8% for the six phenotypes (Fig. 5a) and thus was used in further analysis. FCNN network architecture is illustrated in Fig. 5d. In addition, the utilized FCNN architecture achieved 75.8% accuracy for the testing dataset compared to the 76.7% accuracy for the training dataset, which is 0.9% *training-test gap* which indicates a good ability of the network to generalize to unseen datasets. Also note that increasing the number of hidden layers did not produce measurable improvement, where a 2-hidden layer network produced 75.7% accuracy, as such a single hidden layer FCNN was adopted due to its simplicity and faster classification performance.

To get further insights, in addition to the overall accuracy, we have generated the confusion matrix that showed the classification accuracy for each pair of macrophage phenotypes when all six phenotypes were compared, as depicted in Fig. 5c. It can be noted from the confusion matrix, the true positives of 74.9%, 80.3%, 71.4%, 91.2%, 64.5%, 70.5% respectively for M0, M1, M2a, M2b, M2c, M2d. Consequently, M2b was found to posess the most unique autofluorescence, possibly due to its granularity as noted during optical microscopy. Overall, the lowest confusion was noted between M0, M2b, M1, and M2d, while the highest confusion was between M0, M2c, and M2a. A similarity between M0, M2a, and M2c comparing to M1 were previously reported in gene expression and proteomics studies^51–53^. Our findings suggest that macrophage phenotypes have a detectable unique autofluorescence which allows label-free macrophage identification, however, it should be expected that when simultaneously comparing six phenotypes, some phenotypes are more distinguishable than the others possibly due to the differences in the biological characteristics.

In order to investigate how the overall classification accuracy is affected by the number of classified phenotypes, a combinatorial list was generated for all possible *n*-tuples (where $n\in [2, 6]$). The average accuracy for each *n*-tuple was obtained as reported in Fig. 5b. As anticipated, the classification accuracy decreased with the increasing number of phenotypes in the pool, being 92% for any two label free macrophage phenotypes (pairs), 91.9% for any three (triplets), 84.1% for any four (quadruplets), 80.4% for any five (quintuplets), and 75.8% for all 6 phenotypes as already reported in Fig. 5a. This suggests that macrophage intrinsic autofluorescence signature is a useful feature that can be used to discriminate between six macrophage phenotypes.

## Discussion

Discovering macrophage plasticity is an exciting opportunity for the development of novel diagnostics and therapeutic strategies to manipulate local microenvironment and to support host defense mechanisms. Despite the scientific effort to identify macrophage phenotypical diversity^9,21^, the interactions between macrophages and their local tissue microenvironment remain to be fully elucidated. Current approaches to study macrophage populations are heavily reliant on the identification of cell expressed markers; however, the advances of biomedical tools (e.g., multi-laser flow cytometers and continues spectra flow cytometers), allow the collection of the multiple intrinsic cellular signals emitted during the experiment that can provide additional cell-specific information. This acquired multidimensional data presents a challenge to analyze using manual method; however, applying machine-based computational analysis, in particular neural networks^54^, enables the classification of cell fingerprints in multi-dimensional space.

In this work we showed that six macrophage phenotypes had their unique autofluorescence fingerprints that allowed macrophage label free classification. Firstly, we generated six previously described macrophage phenotypes^11,43,55^ by applying five different stimulations (IFN‐γ and LPS, IL-4, I:C and LPS, IL-10 and TGF‐β, NECA and LPS). Our polarization treatment successfully induced M1, M2a, M2b, M2c, and M2d macrophages from M0 (naïve) as validated using four methods that matched with previous literature reports^11,43,55^: (i) immunostaining was used to observe morphology, (ii) flow cytometry was used to detect cell expressed markers, (iii) arginase and Griess assays were used to determine pathway activated for arginine metabolism. Secondly, a separate set of label-free macrophages were analyzed by a multichannel flow cytometer and the collected data was used to generate phenotype specific signature. Then, a supervised deep learning algorithm was applied for network training and testing, allowing the classification of each macrophage population associated with its unique intrinsic signature. The analysis demonstrated that each macrophage phenotype had a unique autofluorescence fingerprint reveling the potential of using this method for supplementing macrophage phenotyping methods.

To the best of our knowledge this is the first study exploring macrophage phenotyping solely based on autofluorescence characteristics using conventional flow cytometry and machine learning. So far label-free macrophage identification was investigated using microscopy, imaging, and Raman spectroscopy^22–27^. Aside from macrophage identification, there are reports documenting the successful use of autofluorescence to classify other immune cells, for example, inactivated vs activated T-cells using imaging^56^, differentiation of T-cells and CA46 B-lymphoma cells using confocal microscopy^57^, stimulated and unstimulated neutrophils using confocal microscopy^58^. Autofluorescence-based cell sorting of neutrophils and eosins was also demonstrated to yield two pure cell populations with no impact on phenotype and functional characteristics that allowed further investigation post cell sorting^59^. Indeed, imaging and microscopy are great methods to study cell morphology and autofluorescence; however, flow cytometry is equipped with more lasers and therefore broadening the number of detectors allows the discovery of additional features and by that increasing the chances of detecting something unique.

Typically, the investigation of intrinsic cell properties can expand traditional lab methods and add more precision to research findings^11,43,60^. For example, a relevant discovery was reported by Donadon et al.^61^ where the group observed that not overall macrophage density, but the proportion of the morphologically different macrophage subtypes were associated with 5-year disease free survival in metastatic colorectal cancer. Based on these findings they also discovered that these macrophages had unique mRNA signatures^61^. Indeed, macrophage morphology is an important characteristic^52,62,63^ and the understanding of what microenvironmental factors induce such changes in macrophages by applying marker-based methods are very important but requires investments in time and reagents. The proposed framework provides an easy method to investigate macrophage response to polarizing agents, either microenvironmentally derived or therapeutics. In addition, autofluorescence can be very useful to investigate cell populations with shared markers and can potentially be applied in the drug screening and drug discovery.

Since most flow cytometry experiments contain unfixed viability single color control, the proposed investigation of autofluorescence can be easily incorporated to provide an additional experimental data. In particular, it would have a great value to study cell types that cannot be identified via antibody labelling due to the lack of specific markers or reagent availability. In addition to the opportunity to discover novel macrophage phenotypes, we believe that the developed framework could be applied to classify other cell types using flow cytometers with different settings, including spectral flow cytometers. Another application of the proposed methodology is the assistance in flow cytometry panel design as the intensity plot (Fig. 3) can highlight the differences in autofluorescence in each channel and by that can provide a guidance for fluorophore selection.

Although, our method shows a potential to classify macrophage phenotypes by measuring autofluorescence, an important question arises of what cellular characteristics lead to the differences in autofluorescence signatures? It is known that flow cytometers can detect the emission spectra of cell endogenous fluorophores^32^, which are biological molecules like, nicotinamide adenine dinucleotide (NADH), riboflavin, flavin coenzymes, and cell organelles like, mitochondria and lysosomes^23,64,65^. Currently, macrophage phenotypes were shown to have their unique morphology^22^, phenotype specific autofluorescence due to mitochondria organization and metabolic activity^23^, and fluorescent cytoplasmic granules^66^. In addition to the interpretation of autofluorescence signal, future studies are necessary to compare and evaluate different methods, such as microscopy, imaging, flow cytometry, Raman spectroscopy, to identify the best technique or combination of techniques that accurately classify cells based on their intrinsic features. This knowledge can improve our understanding of the unique signatures of different cell populations and the specific features that distinguish them. It will also advance our ability to diagnose diseases, develop new treatments, and understand fundamental biological processes.

To conclude, this study has demonstrated the potential to analyze flow cytometry data by neural networks to reveal the unique signatures for up to six label-free macrophage phenotypes with adequate accuracy. This framework allows a simple, cost-effective, and rapid investigation of changes in cellular autofluorescence in response to the microenvironmental or therapeutic agents, allowing the discovery of novel macrophage phenotypes. In addition, the developed method using macrophages lays a solid platform to investigate and classify other cell types in the future.

## Detailed methods and data analysis

There are three main stages in the developed experiment as depicted in Fig. 1; (i) in the first stage we differentiated six macrophage phenotypes and validated their polarization using standard methods; immunostaining, flow cytometry, arginase and Griess assays, while (ii) in the second stage we performed a flow cytometry experiment where cell intrinsic signals of each unstained macrophage phenotype were collected in all available conventional flow cytometer detectors, (iii) the third stage was performed using computer scripting and involved data importing, machine learning training, and results analysis. The three main stages are explained as follows:

### Stage 1: Macrophage polarization and validation

In order to obtain controllable macrophage phenotypes, we utilized the widely used mouse macrophage cell line RAW 264.7, supplied by the European Collection of Cell Cultures (ECACC; Salisbury, United Kingdom), which was purchased from CellBank Australia (#91,062,702, Westmead, NSW, Australia). The cells were cultured in the growth medium *Dulbecco’s Modified Eagle’s Medium* (DMEM) (Sigma-Aldrich, #D5546), supplemented with 10% heat inactivated fetal bovine serum (FBS) (Thermofisher, #10,100,147), 2 mM L-glutamine (Merck, #G7513), 100 U/mL penicillin and 100 μg/mL streptomycin (Thermofisher, #15,140,122). The cells were maintained by incubating at 37° C temperature with 5% CO_2_ atmosphere. The cells were split after reaching 80% confluency and the total number of passages were limited to 12 to avoid possible genetic mutations.

#### Macrophage Polarization

To generate the six macrophage phenotypes, we sampled the cultured RAW 264.7 cells into 6 well plates with concentration of 0.25 × 10^6^ cells/ml having a total volume of 2 mL of the culturing media. The unstimulated RAW 264.7 were considered as M0 (naïve) macrophages, while M1, M2a, M2b, M2c, M2d polarization states were respectively achieved by applying the following treatments: (i) 25 ng/mL IFN‐γ (Genesearch, #39127S) for 24 h and 25 ng/mL LPS for 24 h were used to generate M1 phenotype; (ii) 25 ng/mL IL-4 (Genesearch, #5208SC) for 24 h to generate M2a phenotype; (iii) 200 µL of immune complexes and 50 ng/mL LPS for 24 h were added to generate M2b phenotype. Immune complexes were made as previously described^67^, briefly, 14 µg of OVA (Sigma-Aldrich, #A5503) was incubated with 39 µL anti-OVA (Sigma-Aldrich, C6534) for 30 min, using rotation at room temperature; (iv) 50 ng/mL IL-10 (Genesearch, #5261SC) and 50 ng/mL of TGF-β (Genesearch, #5231LF ) for 24 h to generate M2c phenotype; (v) LPS 50 ng/mL and 50 μM 5′-(N-thylcarboxamido) adenosine (NECA) (Sigma-Aldrich, #E2387) for 24 h were used to generate M2d phenotype. All polarization experiments were conducted on adherent macrophages, as culturing on the low adherence plates affects macrophage polarization^68^.

#### Polarization Validation Using Immunostaining

Immunostaining was used to demonstrate the morphological changes that occurred in response to polarization agents. For this purpose, RAW 264.7 were polarized in 24 well plate with cell concentration of 5 × 10^5^ cells per well for M0, M2a, M2b, M2c, M2d and 2 × 10^5^ cells per well for M1 phenotype. Attached cells were washed with PBS and fixed with 4% PFA for 20 min at room temperature. After that, the cell membrane was stained by PKH67 (SigmaAldrich, #PKH67GL) as per the manufacturer instructions, and cell nuclei were stained with 0.5 μg/mL DAPI (Abcam, # ab228549) for 5 min at room temperature. Cells were washed again with PBS and the immunofluorescent pictures were captured by FLoid™ Cell Imaging Station (ThermoFisher Scientific) using green FITC (excitation: 482/18 and emission: 532/59) and blue DAPI (excitation: 390/40 (Blue) and emission: 446/33) channels.

#### Polarization Validation using Flow cytometry

We used flow cytometry to determine the changes in the surface and intracellular markers expression. Unpolarized and polarized macrophages were detached and resuspended in MACS buffer (1X PBS, 0.5% BSA, 2 mM EDTA). To prevent antibody unspecific binding, macrophage Fc receptors were blocked with an anti-mouse CD16/CD32 (BD Biosciences, #553,142) for 15 min on ice. For surface staining, cells were stained with mouse anti-CD86, anti-CD69, anti-CD206, anti-TLR1, anti-CD204 for 30 min at 4 °C. For intracellular antigen detection cells were fixed and permeabilized with eBioscience™ Foxp3 / Transcription Factor Staining Buffer Set (eBioscience, #00–5523-00) according to the manufacturer instructions and stained with mouse anti-iNOS for 30 min at room temperature.

In order to detect secreted VEGF and IL-12, brefeldin A (1:1000, Biolegend, #420,601) was added in the final 5 h of polarization to inhibit intracellular protein trafficking. Then after cells fixation and permeabilization, cells were labelled with mouse anti-VEGF and anti-IL-12. All used antibodies we list in a Table 1.

**Table 1:** Reagents used for flow cytometry validation.

Monoclonal antibody	Fluorophore	Clone	Company	Catalogue No
CD86	Brilliant Violet 785	GL-1	Biolegend	105,043
CD69	PE-Cy7	H1.2F3	BD Biosciences	552,879
CD206	Alexa Fluor 700	C068C2	Biolegend	141,734
TLR1	eFluor660	eBioTR23 (TR23)	Thermofisher	50,901,182
iNOS	PE	CXNFT	Thermofisher	12–5920-80
CD204	PE	M204PA	eBioscience	12–2046-80
VEGF	PE	VG1	Novus	NOVNB100664PE
IL-12/IL-23 p40	APC	C15.6	Biolegend	505,205
eBioscience™ Fixable Viability Dye	eFluor™ 780	Thermofisher	65–0865-14	
Viability Dye	DAPI		Abcam	ab228549
	PI		ThermoFisher	P3566

For IL-10 detection, we used Mouse IL-10 Secretion Assay Detection Kit (APC) (MACS, #130–090-939) with manufacturer recommendations followed. Briefly, detached polarized macrophages were incubated with mouse IL-10 catch reagent 1:10 for 45 min at 37 °C. Then cells were labelled with mouse IL-10 detection antibody for 10 min on ice and PI was added to exclude dead cells. In all flow cytometry experiments compensation was performed using Versa comp compensation beads (Beckman Coulter, #B22804 ) and cells were used for the compensation of viability dyes. Cells were analyzed by CytoFLEX LX (Beckman Coulter) and the data analysis was performed using CytExpert (Beckman Coulter), FlowJo v10.8.1(BD), and MATLAB.

#### Polarization validation using arginase assay and griess assay

Arginine metabolism is known to differ between macrophage phenotypes which is also linked to macrophage functional properties. While urea and ornithine are the end products of arginine metabolism in M2 macrophages, nitric oxide is produced in M1 macrophages^43^.

We used arginase assay to measure urea production using previously described protocol^69^. Briefly, 10^6^ polarized cells were lysed by 100 μL 0.1% v/v Triton X-100 in PBS and cells lysate was incubated with 25 mM Tris and 1 mM MnCl_2_ for 10 min at 55 °C. Then, 200 μL of 0.5 M arginine (Sigma-Aldrich, #A1270000) was added and cell lysate was incubated for 1 h at 37 °C. Then we added 900 μL of 44.6N H3PO4, 36N H_2_SO_4_ to stop macrophage arginase enzyme activity and 40 μL 9% (v/v) isonitrosopropiophenone in ethanol (Sigma-Aldrich, #I3502) was added to cell lysate with further incubation for 30 min at 100 °C. Absorbance was measured at 540 nm and urea concentration was estimated using a standard curve generated using twofold serial dilutions of 200 mM urea (200 mM to 3.12 mM) to quantify arginase activity.

Griess assay (Promega, #G2930) was used to measure nitric oxide production and manufacturer recommendations followed. Briefly, 50 μL of supernatant collected from polarized and unpolarized RAW 264.7 was added to 96 well plate. Then samples were incubated in the dark with 50 μL of the sulfanilamide for 10 min, following additional 10 min incubation with 50μL of the N-1-napthylethylenediamine dihydrochloride. Absorbance was measured at 540 nm and nitrite concentrations were estimated using a nitrite standard reference curve generated by twofold dilutions of a 100 μM nitrite solution (100 μM to 1.56 μM).

#### Statistical analysis

Statistical analysis and data visualization were performed using Matlab. The differences between the two groups were compared by using Student’s t-test. Differences with a p value is p > 0.05were considered statistically significant (Fig. 2).

### Stage 2: flow cytometry data collection for unlabeled macrophages

**Figure 6:**
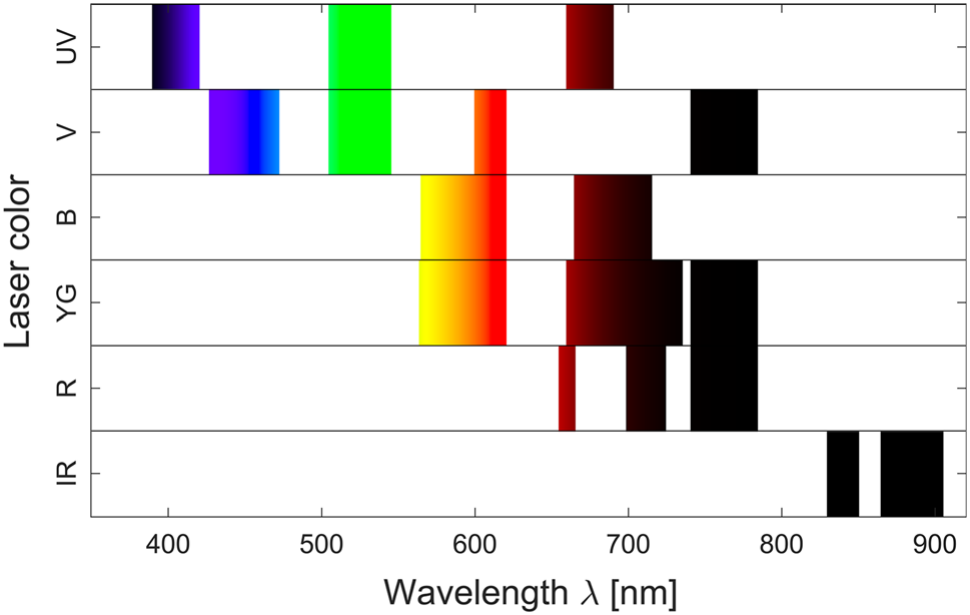
Lasers and detectors configuration of Beckman Coulter CytoFLEX LX machine. The instrument used in this study is equipped with 6 lasers (350 nm ultraviolet, 405 nm violet, 488 nm blue, 561 nm yellow/green, 638 nm red, 808 nm infrared) with each laser having a specific set of optical bandpass filters to collect fluorescence signals.

Cell polarization protocol was followed as per section "Macrophage polarization" with cells counted at harvesting, before and during flow cytometer processing in order to achieve the equal cell numbers per each phenotype. Detached M0, M1, M2a, M2b, M2c, and M2d phenotypes were washed in MACS buffer twice. Then, dead cells were labelled by DAPI, a viability dye that cannot penetrate live cell membrane, at a concentration of 0.5 μg/ml for 10 min on ice, then samples were washed three times with PBS to remove the viability dye. Accordingly, the data was acquired on the Beckman Coulter CytoFLEX LX with signals recorded in all 22 available avalanche photomultiplier detectors using a relative gain of 500, a value known to typically provide optimal dim signal resolution across most fluorescence detectors in the CytoFLEX platform^31^. In total, 45 parameters were recorded out of each cell. The collecting detectors are listed as follows: (i) for the ultraviolet laser (350 nm) the detectors are 405/30, 525/40 and 675/30 detectors; (ii) for the violet laser (405 nm) the detectors are 450/45, 525/40, 610/20, 763/43 detectors; (iii) for the blue laser (488 nm) the detectors are 488/8 (SSC), 525/40, 610/20, 690/50 detectors; (iv) for the yellow/green laser (561 nm) the detectors are 610/20, 763/43, 585/42, 675/30, 710/50 detectors; (v) for the red laser (638 nm) the detectors are 763/43, 660/10, 712/25; and (vi) for the infrared laser (808 nm) the detectors are 840/20 and 885/40. The lasers and associated receiving detectors and bandwidths are indicated in Fig. 6. After acquisition, gated events representing single live cells (DAPI^-^) were exported as .fcs file and analyzed using MATLAB.

### Stage 3: machine learning and results analysis

We used Matlab in our experiment for both machine learning training, data visualization and testing. To train the classifiers, we first exported the results from CytoFLEX LX into a readable format by Matlab, where the data was formatted into a table of 45 columns (corresponding to the 45 aforementioned parameters), and 152,438 rows representing all the single viable cell events captured by the flow cytometer. A separate list of the ground truth *digital labels* was also formed to train the machine and later to test the trained classifiers: this list was based on the pre-known phenotype in each well.

In typical machine learning process, a rule of thumb is to randomly divide the collected data into 60% for machine learning training along with 20% for the validation process, this will leave 20% aside for testing the classifier after the training is done, these are also called *holdout* samples and are not shown to the machine during the training process. To establish a comparison between traditional machine learning methods and neural network, we selected three different machine learning methods as follows:

1. *k*-nearest neighbor (KNN) with 50 neighbors: this is a classical machine learning algorithm aiming to establish a baseline for comparison^48^. The algorithm compares the Euclidian distance between the testing sample vector and the entire training dataset as follows,
  ${d}_{i}=\sqrt{\sum_{j=1}^{45}{\left({v}_{j}-{x}_{ji}\right)}^{2}},$ where $i$ is the index of the cell data entry, $v$ is the testing sample vector, $x$ is the training database, and $j$ is the column index (a total of 45). The algorithm then picks the closest *k* neighbors and assign the predicted class based on the largest population of neighboring classes.
2. Support Vector Machine (SVM); this is another classical machine learning method that is known to produce good results for numerical data types^49^. The algorithm maps training dataset into a feature, i.e., points, in space and tries to maximize the difference between these points. Note that SVM is primarily designed for binary classification problems, however there are well-established methods to extend binary classification into multi-classification. One method is the error-correcting output codes (ECOC) model which builds a codebook and assigns a unique binary code to each class^70^. We adopted this method when testing the SVM for classifying the 6 macrophage phenotypes because it is a built-in Matlab function.
3. Fully connected neural network (FCNN); we used a single wide hidden layer (100 neurons) artificial neural network with one input layer of size 45 (equal to the size of inputs) and one output layer of size 6 (equal to the number of phenotypes)^50^. The network that we utilized is composed of three layers in total, (i) an input layer of 45 neurons matching the number of signals in the dataset, (ii) a hidden layer, where each neuron is connected to every input point from the first layer and to every output point, and (iii) an output layer of 6 neurons matching the number of classes, i.e., the number of phenotypes, each output neuron was connected to all hidden layer neuron as depicted in Fig. 5d. As indicated in Fig. 5, we utilized a single hidden layer architecture of the FCNN due to multiple benefits including: (i) simplicity: a single hidden layer makes the neural network simpler to train, (ii) faster training: with fewer parameters to optimize, a neural network with a single hidden layer can be trained faster than a neural network with multiple hidden layers, (iii) reduced risk of overfitting. As it was shown in the results section, increasing the number of hidden layers did not produce measurable improvements in the classification accuracy.

To avoid classification bias, the 60% training dataset contained almost an equal proportion of each macrophage phenotype (Table 2).

**Table 2:** A proportion of each polarization class used for the training dataset.

M0	M1	M2a	M2b	M2c	M2d
17.03%	17.21%	15.88%	17.63%	15.81%	16.45%

The 20% testing dataset was used to gauge the performance of the three trained classifiers, where the classification accuracy for each classifier was calculated as per the typical method, i.e., Classification Accuracy $=\frac{\sum\nolimits\mathrm{correctly identified cells}}{\mathrm{Total cell number}}$ , this is equivalent to the weighted average of the diagonal elements in the confusion matrix (Fig. 5) where the weighting is according to the number of cells in each class (phenotype).

## Data Availability

All data will be available on reasonable request from the corresponding author.
